# The Influence of Cortisol, Flow, and Anxiety on Performance in E-Sports: A Field Study

**DOI:** 10.1155/2020/9651245

**Published:** 2020-01-28

**Authors:** Steffen C. E. Schmidt, Jens-Peter Gnam, Maximilian Kopf, Tobias Rathgeber, Alexander Woll

**Affiliations:** ^1^Institute of Sports and Sports Science, Karlsruhe Institute of Technology, Karlsruhe, Germany; ^2^Institute of Physical Education and Sports, University of Education, Karlsruhe, Germany

## Abstract

*Background and Objectives*. Most performance theories were tested under controlled laboratory settings and offer therefore only limited transferability to real-life situations. E-sport competitions offer a relatively controllable while at the same time competitive setting, and our aim was to examine different influencing factors on competitive performance. *Design and Methods*. Salivary cortisol was measured immediately before, after, and 30 minutes after a game of 23 computer players during e-sport tournaments. The players answered the Flow Short Scale, which consists of the two subdimensions “flow experience” and “anxiety” subsequent to their game. The performance was assessed by the result of each player's game (win or loss).

**Results:**

Mean cortisol levels increased significantly during the game but response patterns were inconsistent. Winners and losers differed significantly in anxiety with winners showing higher anxiety levels. After dividing the sample into three groups of different cortisol response patterns, significant differences in performance and anxiety were found, with low to moderate levels of cortisol being associated with the highest performance and anxiety.

**Conclusions:**

A low to moderate physiological arousal and a simultaneously high level of anxiety represent a favorable state for achieving optimal performance during e-sports. Anxiety seems to exert a stronger influence on performance than physiological arousal.

## 1. Introduction

The history of theories and models which try to explain the relationship between performance and different influencing factors dates back to the time when Yerkes and Dodson first published their model of an inverted U-shaped relationship between arousal and performance [1]. According to this model, optimal performance should be given when arousal was at a moderate level [1]. When arousal is too low or too high, performance will be inferior [1]. For example, Arent and Landers [2] could clearly demonstrate that the relationship between physiological arousal and performance in a simple response time task followed the proposed inverted U-shaped function.

A further theory ascribing an optimal level of performance to a moderate level of arousal is the flow theory [3–5]. When in a flow state, an individual is able to access its maximal potential and perform at full capacity, while perceiving an optimal level of challenge and arousal without sensed stress [5, 6]. To enter the flow state, two critical prerequisites must be given: (1) an opportunity for action that is perceived as a challenge which engages the person's full skill level (i.e., neither an overload nor an underload) and (2) clear proximal goals and immediate feedback about the progress of goal achievement [6]. Therefore, an important prerequisite for flow is that the task demands are not beyond the person's perceived skills or capabilities [7]. Studies that examined the relationship between markers of physiological arousal and certain aspects of the flow experience predominantly found that a moderate level of physiological arousal corresponds with the highest level of flow experience, resulting in an inverted U-shaped relationship between physiological arousal and flow. For example, Peifer et al. [8] found an inverted U-shaped relationship between physiological arousal (i.e., cortisol and heart rate variability) and flow experience in terms of flow absorption (i.e., being completely immersed in the activity) during a computer task, with moderate levels of physiological arousal correlating with the highest flow levels. Another study could prove that during chess play the highest flow state or flow experience (i.e., flow absorption) correlates with a moderate level of physiological arousal (i.e., cortisol), showing an inverted U-shaped relationship [9]. Furthermore, Tian and colleagues [10] could show that moderate physiological arousal (i.e., heart rate, heart rate variability, and skin conductance) correlates with the highest flow experience, also demonstrating an inverted U-shaped relationship between physiological arousal and flow during playing computer games. In contrast, Keller et al. [11] found that during computer tasks the highest level of physiological arousal (i.e., heart rate variability, and cortisol) corresponds with the highest level of flow experience (i.e., skill-demand-compatibility). However, the problem with these studies is that they are conducted under controlled laboratory conditions and mainly used tasks that were artificially manipulated so that the flow was taken as given when subjects perceived a high level of skill-demand-compatibility. Consequently, group comparisons were almost exclusively done by comparing subjects of different skill-demand-compatibility levels.

A more sophisticated model, which also proposes an optimal relationship between physiological arousal and performance, is the catastrophe model of anxiety and performance [12], which adds anxiety as a further influencing factor of performance. According to this model, the optimal level of performance depends on the interaction of physiological arousal and cognitive anxiety [7]. An increase in cognitive anxiety leads to an enhanced performance when at the same time physiological arousal is low to moderate, but it impairs performance when physiological arousal is high [7]. Furthermore, performance can suddenly drop from a high level to a low level when cognitive anxiety is high and physiological arousal increases, resulting in a performance catastrophe [7]. Thus, optimal performance can be achieved at a high level of cognitive anxiety and a simultaneously low to moderate level of physiological arousal during flow.

Only a few studies have investigated the relationship between physiological arousal, anxiety, flow, and performance at the same time. For example, Hardy and Parfitt [13] found that performances of basketball players were highest during states of high anxiety and low to moderate physiological arousal. Bowlers achieved their best performance in a high anxiety state while being low to moderately physiologically aroused [14]. Furthermore, rock climbers performed better when they were physiologically aroused and when they were anxious compared to when they were not [15]. Duncan et al. [16] found that performance in an anticipation timing test was not negatively affected by high physiological arousal when cognitive anxiety was low, but it deteriorated under high cognitive anxiety and simultaneously high physiological arousal. Of note, in most of these studies, physiological arousal was induced by physical exertion and anxiety was artificially manipulated.

The results of hitherto conducted laboratory studies are not transferable to real-life situations without further ado. To the best of our knowledge, no study has yet explicitly tested these three models and theories under uncontrolled real-life conditions. Hence, we wanted to measure physiological arousal, flow experience, and anxiety as well as the performance itself during a real-life competitive situation without artificially manipulating the underlying conditions (i.e., skill-demand-compatibility, physiological arousal, and anxiety). Therefore, we chose a computer game event, where computer players competed against each other for placement and prices, as an appropriate real-life condition. We hypothesized that (1) playing a computer game during a real-life competition elicits a marked increase in physiological arousal compared to the baseline conditions before the game (cf. [17–19]), (2) winners and losers differ in physiological arousal, flow experience, and anxiety, (3) the relationship between physiological arousal and performance follows an inverted U-form, with moderate levels of physiological arousal corresponding to the best performance (cf. [1, 2]), and (4) the relationship between physiological arousal, anxiety, and performance parallels the catastrophe model of anxiety and performance, with low to moderate levels of physiological arousal and simultaneously high levels of anxiety corresponding to the best performance (cf. [7, 13, 14]).

## 2. Materials and Methods

### 2.1. Participants

The participants were informed about the applied procedures in oral and written forms. All participants gave their written informed consent before participating voluntarily in the study. Participants were free to withdraw from the study at any time without further consequences. The study was conducted in accordance with the Declaration of Helsinki [20] and was approved by the internal review board of the conducting institution. Exclusion criteria for participation in this study were mental health problems and treatment with glucocorticoids due to their impact on measures of interest. At the time of data collection, all participants were in good health and free of any physical and mental complaints. The sample consisted of 19 male and 4 female participants. The characteristics of the participants are displayed in Table 1.

### 2.2. Experimental Approach and Procedure

Playing video games has been found to significantly increase physiological arousal [19]. This effect should be further enhanced by the competitive setting, as competitions have been shown to induce an increase in physiological arousal and anxiety [17] as well as flow [21]. The tournaments took place in the evening between 9 p.m. and 2 a.m. Players could choose between different tournaments in different games (League of Legends and Counter-Strike: Global Offensive). League of Legends is a strategy game in the MOBA (Multiplayer Online Battle Arena) genre and Counter-Strike: Global Offensive is a tactical shooter game, both applying tactical and precision pressure in a real-time setting on the players. The average duration of a player's game was 35 ± 22 minutes. We measured the physiological arousal of the players by collecting cortisol saliva samples since cortisol has been shown to be a valid marker of physiological arousal due to a stress-induced increased activity of the hypothalamus-pituitary-adrenal axis [22, 23]. Baseline cortisol levels were measured immediately before the start of a player's game, immediately after the completion of the game, and 30 minutes afterward. Flow experience and anxiety were assessed using the Flow Short Scale, a questionnaire consisting of two subdimensions that measure flow experience and anxiety individually [24, 25]. The players answered the Flow Short Scale immediately upon ending their game.

### 2.3. Measurements and Instruments

#### 2.3.1. Cortisol

Saliva samples were taken using Salivette® (Sarstedt AG & Co., Nümbrecht, Germany), a synthetic fiber roll, on which the participants had to chew for one minute. After chewing, the saliva samples were put in the plastic container of the Salivette® and stored at −20°C until analysis. Cortisol was analyzed using the Cobas© e 411 analyzer (Roche Diagnostics Germany GmbH, Mannheim, Germany) and applying the electrochemiluminescence technology. Every sample was analyzed in duplicate, and the mean value was then used for the data analysis. Since cortisol levels show a delayed increase in saliva [23, 26], we collected a first postgame saliva sample immediately after the game and a second postgame saliva sample 30 minutes after the completion of the game to capture the stress-induced cortisol peak. We then took the higher one of the two postgame values as cortisol peak for further analysis. However, besides a general cortisol increase from baseline to postgame, our data also showed a cortisol decrease from baseline to postgame in certain subjects. In this case, we took the lower one of the two postgame values as cortisol nadir for further analysis. The participants were instructed not to eat, smoke, drink alcohol or coffee, and refrain from physical activities from one hour prior to their game until the second postgame saliva sample to exclude any influences on cortisol release [27].

#### 2.3.2. Flow Experience and Anxiety

We measured flow experience and anxiety with the Flow Short Scale [24, 25]. Assessing both flow and anxiety is important as flow-inducing challenges are often accompanied by feelings of anxiety or worry [25]. The Flow Short Scale consists of the two subdimensions “flow experience” and “anxiety.” Flow experience is assessed with ten items that cover all the abovementioned components of the flow experience (Cronbach's *α* = 0.90), whereas anxiety is assessed with three items (Cronbach's *α* = 0.80–0.90; [25]). All items are measured on a seven-point Likert scale ranging from 1 to 7. Although flow experience can be further divided into the two factors “absorption” and “fluency,” we operated only with the overall score for flow experience. This is legitimated by the high consistency of the ten flow items.

#### 2.3.3. Performance

The performance of the computer players was assessed by the result of each player's game (i.e., win or defeat). The performance of the different cortisol response groups is then expressed as the ratio of won and lost games among all players in that group.

### 2.4. Data Analysis and Statistics

In order to investigate the difference between baseline and postgame cortisol levels, we calculated a Wilcoxon signed-rank test, since cortisol values were not normally distributed. To compare winners and losers concerning their physiological arousal, flow experience, and anxiety, unpaired *t*-tests were used. We divided the whole sample into three groups, depending on their cortisol response pattern from baseline to postgame (i.e., cortisol decrease (*n* = 6; −3.1 to −0.3 nmol/l), low to moderate cortisol increase (*n* = 10; +0.4 to +5.3 nmol/l), and high cortisol increase (*n* = 7; +10.6 to +13.2 nmol/l)). The two groups showing a cortisol increase were divided at the mean. The groups were then compared concerning their flow experience and anxiety levels using one-way ANOVA. We adjusted the significance level for the post hoc tests using Bonferroni corrections to account for multiple tests with the same sample. The level of significance was set at *p* < 0.05 for the two-sided tests. Effect sizes were calculated as Cohen's *d* for the *t*-tests and the Wilcoxon signed-rank test and as partial eta-squared (*η*_*p*_^2^) for ANOVA. All analyses and statistics were performed with the IBM SPSS Statistics 24 software (IBM Corp., Armonk, USA).

## 3. Results

The average cortisol level of all participants increased significantly from baseline to postgame conditions, demonstrating a strong effect and constituting a marked physiological arousal (3.5 ± 2.2 versus 7.7 ± 6.3 nmol/l; *z* = −2.95, *p* < 0.01, *d* = 1.56).

Winners and losers only differed significantly concerning their anxiety level (*t* = 3.80, *p* < 0.01, *d* = 1.58), with winners showing higher anxiety levels (5.5 ± 1.6 versus 3.2 ± 1.3). Concerning cortisol and flow levels, the differences were not significant (see Table 2).

Looking at the three groups with different cortisol response patterns, the performance was best in low to moderately aroused players (mean cortisol change: +2.3 ± 1.8 nmol/l), as they won most of their games (wins–defeats: 7–3; ratio: 2.3). Highly aroused players (mean cortisol change: +11.8 ± 1.0 nmol/l) performed second best (wins–defeats: 4–3; ratio: 1.3), while players with decreasing physiological arousal (mean cortisol change: −1.5 ± 1.1 nmol/l) performed worst (wins–defeats: 1–5; ratio: 0.2). Comparisons between those groups of different cortisol response patterns revealed no significant differences concerning their flow experience (*F*_2,20_ = 0.44, *p*=0.65, *η*_*p*_^2^ = 0.04). However, these groups differed significantly in regard to their anxiety levels (*F*_2,20_ = 6.72, *p* < 0.01, *η*_*p*_^2^ = 0.40), demonstrating a strong effect. Post hoc tests revealed a significant difference in anxiety between the group showing a cortisol decrease response and the group showing a low to moderate cortisol increase response (*p* < 0.01). The descriptive statistics are displayed in Table 3.

## 4. Discussion

Playing computer games under competitive real-life conditions significantly increased the mean cortisol level and thus physiological arousal of the sample. Therefore, our first hypothesis could be confirmed and our results are in line with earlier studies, also demonstrating marked physiological arousal when subjects played computer games or participated in sports competitions [17–19]. However, eminently there were different response patterns among participants and it is striking that, among those six participants with a decrease in cortisol level, five lost their games and one was a semiprofessional e-sports athlete who was measured during his finals on the stage which he won. Here, the prerequisite for flow and physical arousal stated by Hardy [7] that the task demands should not be beyond the person's perceived skills or capabilities [7] may have been violated. This could also explain the results from other e-sport related pilot studies that found no significant increase in cortisol levels playing League of Legends [28].

As winners and losers only differed significantly in anxiety but not ultimately in physiological arousal and flow in our study, our second hypothesis could only be partially confirmed. A lack of difference in flow between winners and losers may be due to the fact that, in our real-life setting, each and every participant had a relatively high level of flow (see Table 3), and since we have chosen a real-life setting, we did not artificially manipulate the skill-demand-compatibility which is crucial for flow [7]. Post hoc power analyses showed that, given our sample size, only group differences in flow experience of *d* = 1.28 or 1.42 can be precluded with a power of *β* = 0.80. Studies that confirmed an inverted U-shaped relationship between physiological arousal and flow experience manipulated the skill-demand-compatibility of their experimental groups so that these groups differed significantly in their flow experience [8–10]. Hence, it seems that anxiety has a greater influence on real-life performance in e-sports since flow is almost always given. A lack of difference in physiological arousal between winners and losers in mean our study as shown in Table 3 may be simply due to the fact that, according to the law of Yerkes and Dodson [1], the relation between arousal and performance is not linear.

**Figure 1:**
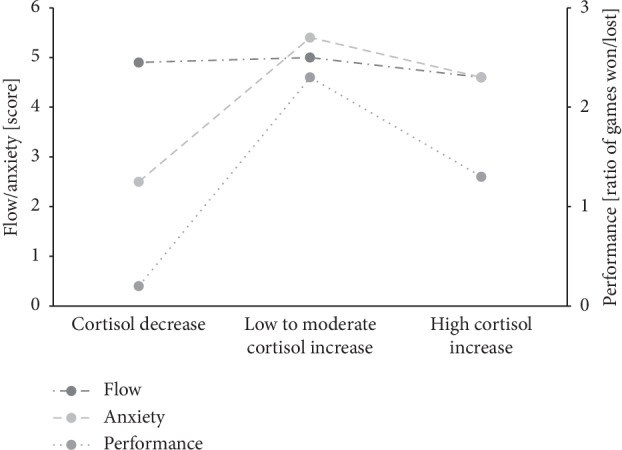
Flow, anxiety, and performance levels of groups with different physiological arousal patterns.

Therefore, we stratified our sample in groups with different physiological arousal patterns and those results demonstrate an inverted U-shaped relationship for physiological arousal and performance (see Figure 1). The group with a low to moderate cortisol increase achieved the highest performance, whereas the group with a high cortisol increase performed second best and the group showing a cortisol decrease performed worst (see Table 3). These results confirm our third hypothesis and the results of Yerkes and Dodson [1] as well as the recent research on physiological arousal and performance by Arent et al. [2, 29] and therefore validated the Yerkes–Dodson law for the real-life situation of competitively playing real-time computer games with high tactical and precision pressure. Nevertheless, the fact that we encountered arousal nonresponders with a pre- to postgame decrease in cortisol clearly shows that participating in gaming and e-sports does not guarantee high physiological arousals and therefore is not clearly comparable to other highly physical sports. Further studies with larger samples that take the level of gaming experience into account are needed to substantiate these findings.

Regarding our fourth hypothesis, groups with different physiological arousal patterns differed significantly concerning their anxiety level. The group with a low to moderate cortisol increase showed the highest anxiety levels, whereas the group with a cortisol decrease showed the lowest anxiety levels (see Table 3). The results represent an inverted U-shaped relationship between physiological arousal and anxiety, with low to moderate physiological arousal corresponding to the highest anxiety (see Figure 1). As the relationship between physiological arousal and performance also demonstrated an inverted U-shaped function, we can draw the conclusion that a low to moderate level of physiological arousal and a simultaneously high level of anxiety result in the best performance. These results are in line with the catastrophe model of anxiety and performance and with the results of former studies [7, 13, 14]. This confirms the catastrophe model of anxiety and performance. Interestingly, although our groups showed no significant difference in flow experience, flow levels are indeed the highest in the group with a low to moderate physiological arousal and with the highest anxiety and performance levels (see Table 3). One can now only speculate whether different skill-demand-compatibilities among the players would have led to significant differences in flow experience.

The benefit to the current body of knowledge from our study is that it provides results from a real-life situation that reaches beyond what is hitherto known from the laboratory studies. By choosing e-sports as an example for a real-life competition, we could also exclude some confounding variables that typically occur during physical sports competitions and affect the cortisol response like dehydration, hypoglycemia, extreme muscular and/or metabolic stress, and the consumption of food or drinks during the competition. Professional as well as semiprofessional and recreational e-sport is a growing field and sport science pays increasing attention to it [30]. Nevertheless, there are also some critical aspects of our study. First of all, the relatively small sample size limits the generalization of the results. Moreover, it would have been useful to collect cortisol samples throughout a prolonged recovery period. This would have provided information on individual recovery from physiological arousal. Unfortunately, this study's setting (the least interference possible) precluded us from collecting more than two samples after a player's game. Concerning the assessment of the players' performance, it would have been interesting to have further variables and parameters to analyze performance in more detail. But since the available game data in the played games are heavily dependent on the specific opponent, the specific role of the player, and/or the length of the game, the game result (i.e., win or defeat) and the ratio of games won and games lost constituted the best available performance parameter.

## 5. Conclusion

This study could demonstrate that playing computer games in a competitive real-life situation can result in significant physiological arousal expressed by an increase in cortisol levels. The relationship between physiological arousal and performance demonstrates an inverted U-shaped function, with low to moderate physiological arousal resulting in the best performance. We also found an inverted U-shaped relationship between physiological arousal and anxiety. Physiologically aroused and anxious subjects performed much better compared to subjects who were not physiologically aroused and not anxious independent of their respective flow levels. We could validate the Yerkes–Dodson law as well as the catastrophe model of anxiety and performance for the real-life situation of competitively playing computer games. Overall, higher levels of anxiety seem to be beneficial for e-sport performance and constitute a more significant influencing factor of e-sport performance than physiological arousal or flow experience.

## Figures and Tables

**Table 1:** Descriptive statistics of the sample.

Characteristic (*n* = 23)	M ± SD	Minimum	Maximum
Age (years)	23.7 ± 3.3	20.0	33.0
Height (cm)	176.7 ± 8.3	161.0	189.0
Weight (kg)	76.8 ± 15.1	54.0	115.0
BMI (kg/m^2^)	24.6 ± 4.3	15.1	35.5
Cortisol baseline (nmol/l)	3.5 ± 2.2	1.5	10.7
Cortisol postgame (nmol/l)	7.7 ± 6.3	1.5	23.9
Flow	4.8 ± 0.9	2.9	6.4
Anxiety	4.4 ± 1.9	1.3	7.0

**Table 2:** Descriptive and inferential statistics according to performance.

Dependent variables (mean ± standard deviation)	Game won (*n* = 12)	Game lost (*n* = 11)	*t*-test
Age (years)	23.5 ± 3.0	24.0 ± 3.8	*t* = −0.35, *p*=0.73
Height (cm)	176.5 ± 8.4	176.8 ± 8.5	*t* = −0.09, *p*=0.93
Weight (kg)	71.2 ± 13.5	82.5 ± 15.1	*t* = −1.85, *p*=0.08
BMI (kg/m^2^)	22.6 ± 2.3	26.5 ± 5.0	*t* = −2.37, *p*=0.03^*∗*^
Cortisol baseline (nmol/l)	3.2 ± 1.9	3.7 ± 2.4	*t* = −0.50, *p*=0.63
Cortisol postgame (nmol/l)	8.2 ± 5.5	7.1 ± 7.2	*t* = 0.40, *p*=0.70
Cortisol difference (nmol/l)	4.9 ± 5.4	3.4 ± 5.8	*t* = 0.64, *p*=0.52
Flow	5.0 ± 1.0	4.6 ± 0.7	*t* = 0.98, *p*=0.33
Anxiety	5.5 ± 1.6	3.2 ± 1.3	*t* = 3.80, *p* < 0.01^*∗*^

^*∗*^Significant difference.

**Table 3:** Descriptive statistics (mean ± standard deviation) of groups with different cortisol response patterns.

Dependent variables (mean ± standard deviation)	Cortisol decrease (*n* = 6)	Low to moderate cortisol increase (*n* = 10)	High cortisol increase (*n* = 7)
Age (years)	23.8 ± 4.6	24.1 ± 3.3	23.1 ± 2.5
Height (cm)	179.7 ± 5.6	177.8 ± 9.3	172.4 ± 7.9
Weight (kg)	90.0 ± 16.6	78.5 ± 10.6	65.0 ± 11.6
BMI (kg/m^2^)	27.5 ± 4.6	24.9 ± 3.4	22.0 ± 4.2
Cortisol baseline (nmol/l)	3.6 ± 1.2	3.0 ± 2.0	4.2 ± 3.0
Cortisol postgame (nmol/l)	2.1 ± 0.6	5.3 ± 2.8	16.0 ± 3.6
Flow	4.9 ± 0.6	5.0 ± 0.8	4.6 ± 1.2
Anxiety	2.5 ± 0.7	5.4 ± 1.9	4.6 ± 1.4
Performance (wins–defeats/ratio)	1 – 5/0.2	7 – 3/2.3	4 – 3/1.3

## Data Availability

The data used to support the findings of this study are available from the corresponding author upon request.

## References

[1] Yerkes R. M., Dodson J. D. (1908). The relation of strength of stimulus to rapidity of habit-formation. *Journal of Comparative Neurology and Psychology*.

[2] Arent S. M., Landers D. M. (2003). Arousal, anxiety, and performance: a reexamination of the inverted-U hypothesis. *Research Quarterly for Exercise and Sport*.

[3] Csikszentmihalyi M. (1975). *Beyond Boredom and Anxiety: Experiencing Flow in Work and Play*.

[4] Csikszentmihalyi M. (1990). *Flow: The Psychology of Optimal Experience*.

[5] Jackson S. A., Csikszentmihalyi M. (1999). *Flow in Sports: The Keys to Optimal Experiences and Performances*.

[6] Nakamura J., Csikszentmihalyi M., Snyder C. R., Lopez S. J. (2009). The concept of flow. *Oxford Handbook of Positive Psychology*.

[7] Hardy L. (1999). Stress, anxiety and performance. *Journal of Science and Medicine in Sport*.

[8] Peifer C., Schulz A., Schächinger H., Baumann N., Antoni C. H. (2014). The relation of flow-experience and physiological arousal under stress—can u shape it?. *Journal of Experimental Social Psychology*.

[9] Tozman T., Zhang Y. Y., Vollmeyer R. (2017). Inverted U-shaped function between flow and cortisol release during chess play. *Journal of Happiness Studies*.

[10] Tian Y., Bian Y., Han P., Wang P., Gao F., Chen Y. (2017). Physiological signal analysis for evaluating flow during playing of computer games of varying difficulty. *Frontiers in Psychology*.

[11] Keller J., Bless H., Blomann F., Kleinböhl D. (2011). Physiological aspects of flow experiences: skills-demand-compatibility effects on heart rate variability and salivary cortisol. *Journal of Experimental Social Psychology*.

[12] Fazey J. A., Hardy L. (1988). *The Inverted-U Hypothesis: A Catastrophe for Sport Psychology*.

[13] Hardy L., Parfitt G. (1991). A catastrophe model of anxiety and performance. *British Journal of Psychology*.

[14] Hardy L., Parfitt G., Pates J. (1994). Performance catastrophes in sport: a test of the hysteresis hypothesis. *Journal of Sports Sciences*.

[15] Hardy L., Hutchinson A. (2007). Effects of performance anxiety on effort and performance in rock climbing: a test of processing efficiency theory. *Anxiety, Stress, & Coping*.

[16] Duncan M. J., Smith M., Bryant E. (2016). Effects of increasing and decreasing physiological arousal on anticipation timing performance during competition and practice. *European Journal of Sport Science*.

[17] Clarke E., Sagnol M., Ferrand C., Maso F., Lac G. (2001). Psychophysiological stress in judo athletes during competitions. *Journal of Sports Medicine and Physical Fitness*.

[18] Segal K. R., Dietz W. H. (1991). Physiologic responses to playing a video game. *Archives of Pediatrics & Adolescent Medicine*.

[19] Wang X., Perry A. C. (2006). Metabolic and physiologic responses to video game play in 7- to 10-year-old boys. *Archives of Pediatrics & Adolescent Medicine*.

[20] World Medical Association (WMA) (2013). World Medical Association Declaration of Helsinki: ethical principles for medical research involving human subjects. *Journal of the American Medical Association*.

[21] Chen J. (2007). Flow in games (and everything else). *Communications of the ACM*.

[22] Axelrod J., Reisine T. (1984). Stress hormones: their interaction and regulation. *Science*.

[23] Kirschbaum C., Hellhammer D. H., Fink G. (2000). Salivary cortisol. *Encyclopedia of Stress*.

[24] Engeser S., Rheinberg F. (2008). Flow, performance and moderators of challenge-skill balance. *Motivation and Emotion*.

[25] Rheinberg F., Vollmeyer R., Engeser S., Stiensmeier-Pelster J., Rheinberg F. (2003). Die erfassung des flow-erlebens (the assessment of flow experience). *Diagnostik von Motivation und Selbstkonzept*.

[26] Dickerson S. S., Kemeny M. E. (2004). Acute stressors and cortisol responses: a theoretical integration and synthesis of laboratory research. *Psychological Bulletin*.

[27] Nicolson N. A., Luecken L. J., Gallo L. G. (2008). Measurement of cortisol. *Handbook of Physiological Research Methods in Health Psychology*.

[28] Gray P. B., Vuong J., Zava D. T., McHale T. S. (2018). Testing men’s hormone responses to playing league of legends: No changes in testosterone, cortisol, DHEA or androstenedione but decreases in aldosterone. *Computers in Human Behavior*.

[29] Lautenbach F., Laborde S., Achtzehn S., Raab M. (2014). Preliminary evidence of salivary cortisol predicting performance in a controlled setting. *Psychoneuroendocrinology*.

[30] Cunningham G. B., Fairley S., Ferkins L. (2018). eSport: construct specifications and implications for sport management. *Sport Management Review*.

